# Reproductive capital: theoretical foundations and empirical evidence from the workplace

**DOI:** 10.3389/fsoc.2025.1608368

**Published:** 2025-08-04

**Authors:** Robin A. Hadley, Clare Mumford, Michael Carroll, Krystal Wilkinson

**Affiliations:** ^1^Department of Life Science, Manchester Metropolitan University, Manchester, United Kingdom; ^2^Institute for Research Into Organisations, Work and Employment, University of Central Lancashire, Preston, Lancashire, United Kingdom; ^3^Business School, Faculty of Business and Law, Manchester Metropolitan University, Manchester, United Kingdom

**Keywords:** reproductive capital, assisted reproductive technologies (ART), infertility, workplace polices and fertility, Bourdieu's theory of capital, symbolic violence, economic eugenics, rhizomatic relationships

## Abstract

**Introduction:**

The workplace encompasses structural and personal tensions related to both reproduction and non-reproduction, as well as ambiguity and ambivalence permeate policies, practices, and social interactions. The aim of this study was to explore and record participants' diverse fertility journeys and the effect of work on their preconception/infertility experience.

**Method:**

The concept of “reproductive capital” emerged from a latent thematic analysis of semi-structured bio-narrative interviews with 80 people (67 women and 13 men) and their accounts of how the workplace affected their reproductive journeys.

**Results:**

The workplace was an alienating space for people accessing Assisted Reproductive Technology (ART) treatment, who were subjected to scrutiny and judgment. The challenges faced by individuals included matters surrounding disclosure and subsequent consequences, desired/expected support, and what was received. Some participants challenged the pronatalist policy status quo and sought change to make the workplace more inclusive. Participants' accounts highlighted numerous situations in which reproductive capital was present.

**Discussion:**

Contextualizing theory through empirical data allows for a broader understanding of how socio-economic, socio-cultural, norms, and values influence individual and organizational behavior. This article critically examines the concept of “reproductive capital” and its interaction with other forms of capital: aging, biological, cultural, economic, social, and symbolic.

## 1 Introduction

In this article, we explore the findings of a 2-year research project (2020–22) examining the intersection of people's experiences of Assisted Reproductive Technology (ART) and the workplace (Wilkinson et al., 2022). This is an extremely sensitive subject: this piece includes people's experience of bereavement and loss that may trigger upset.

Over the last 50 years, a demographic trend of reduced fertility rates, postponed parenthood, and an increase in childlessness and age of mortality has been observed in both the global North and South, with significant variations within and between countries (Yopo Díaz and Watkins, 2025). In Europe, one in four men and one in five women are childless (Kreyenfeld and Konietzka, 2017, p. 10), with the majority being childless by circumstance (Archetti, 2020). Alongside, this demographic shift is an increase in ART availability. ART has become a global industry, with over eight million *in vitro* fertilization (IVF) babies born (Inhorn, 2020, p. 48) and treatment centers in 132 countries [International Federation of Fertility Societies' Surveillance (IFFS), 2022]. ART has been identified as an across-the-board speculative equity investment (Van De Wiel, 2020), with the global IVF market projected to reach $37.4 billion by 2030 (Grand View Research, 2022). The fertility industry has expanded to form a global trade of currency, people, sex cells, skills, technology, and transnational commercial surrogacy (TCS; Rudrappa, 2015; Jacobson, 2016; Smietana et al., 2021; Tober, 2024).

In addition to treating medical infertility, ART provides a route to biological parenthood for people who previously had been excluded from this status, such as single men and women, same-sex couples (Inhorn, 2020; Wilkinson et al., 2023), and for some career reasons (e.g., actors, models, musicians, politicians; Cutajar, 2022). Financing fertility treatment varies from country to country, and includes full state support, company-sponsored fertility benefits (often only available to professional classes, including egg-freezing), health insurance, individual finance, and a mix of arrangements (Inhorn, 2020; Mackenzie et al., 2024). Nonetheless, many countries limit subsidies for ART, with marginalized groups less likely to receive funded treatment. Many of those who pursue parenthood via ART face complex decisions on how to find the means (capital) to pay for treatment, from their own financial resources, familial support, borrowing (loans, mortgages), or trading (egg donation to offset treatment costs). Critics argue that the high cost of ART limits access to those who can afford it (Tober, 2024; Yopo Díaz and Watkins, 2025) and a contemporary development of historical biopolitical eugenic policies of planned selection and reduction of the population of those deemed economically, intellectually, physically, racially, and socially “lesser” (Roberts, 1997; Rembis, 2009; Rudrappa, 2015; Rutherford, 2022).

A key theme of the study was the relationship between ART and the participants' economic situation and how these intersected with their respective sociocultural situations. This led us to view our data using Bourdieu's (1986) theoretical tools of capital, habitus, and field which provide a multidimensional perspective for understanding social dynamics, individual positioning within society, and internalized dispositions. Using a Bourdieusian lens resulted in the formulation of “reproductive capital” (RC) as a way of identifying the environmental, individual, social, and structural conditions surrounding an individual's resources and capacities in relation to reproduction. This includes biological factors such as fertility, as well as social, cultural, and economic resources (capital) that enable or constrain reproductive choices and outcomes. RC intersects with other forms of capital (economic, social, and cultural) and shapes one's status and experience in various social fields, including the workplace. By employing Bourdieusian concepts (1977, 1984, 1986) of capital, habitus, and field (see Section 2.3), we believe that RC provides an important new framework for understanding the complex rhizomatic relationships between reproduction, individual and cultural environments, and societal structures.

We identify RC as embodied in individual agency and embedded in an organizational structure in three principal areas. First, the workplace is a site where biological, cultural, economic, human, reproductive, social, and symbolic capital intersect, and where identities and potential are contested (Hadley, 2021a; Wilkinson et al., 2023). For the individual, entry and occupational progression require specific forms of capital (e.g., appearance, language, qualifications) reflecting sociocultural norms (Bourdieu, 1984). Likewise, it is a location where reproductive intentions and outcomes interact with micro, macro and meso policies, practices and legislation. Second, biological capital and RC interact with other forms of capital, and there is a continuum of responses to individuals' past, present, and/or future reproductive identity, ranging from socioeconomic and cultural support and celebration of and for successful reproduction to ambiguity, ambivalence, and stigmatization (Letherby, 2016; Tsigdinos, 2022; Zhang and Soderberg, 2023) that surrounds non-reproduction, unwanted reproduction, and/or unwlecome parenthood (Ramsay and Letherby, 2006; Power, 2014; Donath, 2017; Hall, 2022; Yopo Díaz and Watkins, 2025). The third is the negotiation of age-related societal norms (biological and social (biosocial) clocks; reproductive careers) regarding the appropriate age and timing of parenthood (Billari et al., 2011; Goldberg, 2014; Johnson et al., 2018; Yopo Díaz, 2021a,b; Wilkinson and Rouse, 2023). The remainder of this paper is organized into five parts. The first provides an overview of reproduction, followed by an outline of the theoretical position employed. The third section describes the methods undertaken in the study: a qualitative exploration of how people experience the interactions involved in their fertility journey(s) and employment. The fourth part draws on empirical data to explore RC and economic, biological, social, and symbolic capital and the workings of symbolic violence. Finally, the discussion section examines the concept of RC and highlights the limitations of the study.

## 2 Background and theoretical framework

Historically, despite their inherent rhizomatic interconnections, social science research on fertility and infertility has operated as a two-siloed approach in which people's “reproductive events” (abortion, intended/unintended pregnancy, perinatal loss) are measured as discrete outcomes. Consequently, people's lived experiences and embodied and interconnected environmental, social, and structural factors beyond the biomedical field were excluded (Johnson et al., 2018, 2023). Feminist scholars and activists have shown that the gendered nature of the dominant biomedical model of infertility and cultural norms around reproduction disproportionately burdened women and marginalized men (Mason, 1993; Inhorn et al., 2009; Lohan, 2015; Letherby, 2016; Yopo Díaz and Watkins, 2025). Feminist research highlights the profound effect of infertility on men's self-perception and societal role, with men's fertility issues rarely explored in the literature on masculinity (Throsby and Gill, 2004; Daniels, 2006; Hammarberg et al., 2017). Reproductive rights are at the core of feminisms and have been central to the development of critical concepts (Tilley et al., 2012), such as social reproduction (Katz, 2001; Power, 2014), stratified reproduction (Colen, 1995; Ginsburg and Rapp, 1995), reproductive justice (Ross and Solinger, 2017; Saluk, 2024), reproductive identity (Becker, 1999; Athan, 2020) and reproductive careers (Johnson et al., 2018, 2023). The latter is a life-course framework that bridges the research silo by accounting for the biological and social processes that encompass people's past, present, and future reproductive experiences, attitudes, and behaviors: their reproductive careers. All these approaches illustrate how reproductive practices and events, such as abortion, contraception, infertility episodes, intended or unintended pregnancies, live births, and perinatal loss, are interconnected and contingent (Johnson et al., 2018, 2023; Katz, 2001; Power, 2014; Hall, 2022). These reproductive frameworks are important for understanding how reproduction is situated within specific social and economic contexts (Greenhalgh, 1995) at the micro, meso, and macro levels (Katz, 2001; Power, 2014; Hall, 2022). In this piece, we add a new perspective to comprehend the intricate intersections of economic and sociocultural environments, inequality, knowledge, power, reproduction, and technology in people's reproductive journeys.

The gendered nature of infertility research and cultural norms surrounding reproduction are manifested in the wider pronatalist heteronormative ideology of the “motherhood mandate” (Russo, 1976, p. 144), women, and “the package deal” for men of work, relationship, and fatherhood (Townsend, 2002) influencing their reproductive careers (Johnson et al., 2018, 2023). Biological continuity is a respected adult status conferring a valued social identity that entails duties, kudos, privileges, responsibilities, and rights throughout life (Dykstra and Hagestad, 2007). Parenthood is considered fundamental in the “normal, expectable life-cycle” (Neugarten, 1969, p. 125). All major religions perpetuate the child-producing ideal: children are viewed as a blessing and childlessness stigmatized as “barrenness” and “unnatural” (Letherby, 2016). Little attention has been paid to the preconception or non-reproductive bodies of women (Ramsay and Letherby, 2006; Wilkinson et al., 2023) and less so for men (Zaake et al., 2019; Hadley, 2020). Ginsburg and Rapp (1995, pp. 3–4) argue women's reproductive experiences (e.g., pain, risk, and harm) have been discursively excluded and—through “euphemized violence”—replaced by a “beneficent discourse” including a “pernicious effacement” of men's experiences. People who do not fulfill the parental ideal are frequently subject to “stigmatization, stereotyping, discrimination, exclusion, isolation, and mistrust” (Hadley, 2019, p. 53).

Parenthood is a central tenet of pronatalism and is constructed as a natural, spontaneous, and unconscious act that is unreflectively and unquestionably accepted/expected (Morison, 2013). Pronatalism encompasses political, religious, and cultural attitudes that encourage reproduction and idealize parenthood (Bajaj and Stade, 2022). These ideologies are pervasive across cultural, ideological, and psychological dimensions at all policy levels (Heitlinger, 1991). Consequently, pronatalism is a global phenomenon that affects workplaces, family life, relationships, health and social care, and leisure environments (Van Balen and Inhorn, 2002; Ramsay and Letherby, 2006). Nonetheless, many people's experiences of parenthood do not match the “ideal” (Fox and Quinn, 2014; Archetti, 2020). The romanticisation of childbirth and motherhood masks the physiological and psychological trauma experienced by many women (Busari and Nwafor, 2023). Many studies have reported that parenting is associated with increased depression and stress and decreased wellbeing (Rizzo et al., 2013). Donath (2015) argues that regret should be included in the transition to motherhood. The highly contextual nature of reproduction (Greenhalgh, 1995) underscores the structural intersectionality perpetuating reproductive bias and discrimination (Johnson et al., 2023), elucidating how ableist, classist, and racist stereotypes are ingrained in law, politics, policy, media, social narratives, and welfare, manifesting as emotional, physical, and verbal mistreatment (termed “obstetric violence”) during the perinatal period (Fox and Quinn, 2014; Chadwick, 2019). Consequently, scholars and activists have identified how reproductive discourse links oppression through health and welfare policies that disproportionally affect black, disabled, indigenous, marginalized, migrant, minority, and low-income people (Hall, 2022; Yopo Díaz and Watkins, 2025). For example, people are excluded from fertility services because of class, citizenship, economics, gender, and/or race (Smietana et al., 2018; Yopo Díaz and Watkins, 2025).

### 2.1 Fertility

Globally, the World Health Organization (World Health Organization, 2023b) estimates that one in six people experiences infertility at some point in their lives. Infertility is a disease of the reproductive system that affects both men and women: “infertility does not discriminate” (World Health Organization, 2023a). It is defined as the inability to achieve conception after 12 months or more of regular, unprotected sexual intercourse. Diagnosis of infertility can result in considerable distress culturally, economically, mentally, and physically, and socially (including stigma). Childlessness has often viewed as two distinct categories: “voluntary” and “involuntary.” The latter is often associated with unsuccessful infertility treatment (Hadley, 2019). Similarly, parental status was viewed as a binary of childless vs. parent, but contemporary scholars argue for a “continuum of parental statuses” (Albertini and Kohli, 2017) reflecting fluidity in family practices (Morgan, 2011). The continuum includes various reproductive identities: biological parents, childless-by-circumstance, childfree, chosen childless, incel, infertile, involuntarily childless, mediated childless, social, or stepparent (Letherby, 2016; Athan, 2020). People's locations on this continuum may change at any time during their lives, depending on a range of factors: environmental, macro socioeconomic, and/or micro personal (Blackstone and Stewart, 2012; Power, 2014). For example, some people choose not to have children to avoid passing on hereditary conditions or chromosomal anomalies (Kelly, 2009) while involuntary celibates (incels) view the “mating market” as anxiety inducing, with the cost of rejection outweighing any benefit (Whittaker et al., 2024).

In the UK, the National Health Service (NHS) ART service acceptance criteria are geographically variable, leading people to move to access treatment (Wilkinson et al., 2023). Many people draw on their economic resources (often tens of thousands of pounds) to access private treatment. ART is portrayed as a highly effective treatment, despite substantial evidence that gamete loss and miscarriage are extremely common (Littleton and Bewley, 2019; Tsigdinos, 2022; Van Balen and Inhorn, 2002). In the UK, 76% of IVF embryo transfers did not result in a live birth (Human Fertilisation and Embryology Authority, 2024). Many individuals with prenatal and natal loss experience grief, mental and physical ill-health, poor wellbeing, and social and economic stress (Bueno, 2019; Jones et al., 2019). Historically, fathers have been excluded from miscarriage studies (Oakley et al., 1990) and recent studies have demonstrated the impact of prenatal, pregnancy, and neonatal losses on men (Bueno, 2019; Hadley, 2021a; Jones et al., 2019). These are significant in reflecting the sociocultural disenfranchisement of childless people: many involuntarily, circumstantially, and chosen childless people report feeling outsiderness, shame, and stigmatized (Exley and Letherby, 2001).

### 2.2 Reproduction, career, and workplace dynamics

Globally, there is a wide range of workplace protections and provisions related to maternity and parenting, although they vary and are not always observed. In the UK, for example, “pregnancy and maternity” is a “protected characteristic” under the Equality Act (2010), providing various rights, including protection from discrimination. By contrast, few countries have protections and provisions for those undergoing ART with Wilkinson et al. (2023, p. 6) arguing, “employer ART-related supports usually rests on business case cost-benefit calculation.” A minority of employers have developed policies and provisions for employees undergoing ART. However, when policies are in operation, only certain types of workers are covered; they often fail to consider the needs of partners and diverse family identities and fail to account for the temporalities and diverse outcomes of complex fertility journeys (Wilkinson et al., 2023).

### 2.3 Theoretical framework

To understand the complex social dynamics surrounding participants' experiences of ART and work, we utilized Bourdieu's (1977; 1984; 1986; 1987; 1996; 2002) theoretical tools of capital, habitus, and field which allow for analysis at both the structural and individual levels while highlighting issues of inequality, power, and social norms. Bourdieu (1986) identified four types of capital: economic (income, wealth, assets), social (networks, relationships), cultural (education, speech, taste), and symbolic (prestige and status). Cultural capital manifests as institutionalized (education), materialized (arts, books), and embodied (behavior and thoughts). Habitus refers to internalized social structures that shape individual dispositions and identities (Mullins, 2018a). It links social structures with personal emotions and shows internalized practices (Reay, 2015, p. 21). Elliott (2009, p. 147) argues habitus includes “cultural tastes and social preferences” as power and class demonstrations. There is a dynamic relationship between habitus and embodied capital—“embodied cultural capital” (Garrett, 2016, p. 82)—highlighting distinctions in accent, gait, gaze, gesture, and posture. “Fields” are social spaces for interactions to exchange capital (artistic merit, skill, money, prestige), power, and status, reflecting cultural, economic, political, and social contexts (Kilvington-Dowd and Robertson, 2020, p. 21). As a field, the workplace can create social capital that offers emotional, financial, and practical benefits (Urry, 2007, p. 198), yet it may “enable or constrain choices on identity expression in specific cultural contexts” (Simpson, 2013, p. 297; Sharma and Samanta, 2020). For instance, in China, the social capital of “guanxi” (work-based intimate relationships, Qi, 2013) features strategies for material benefits (Tang, 2020).

Bourdieu (1996, p. 23) argued that “family” and “the centrality of the mother” were key in cultural capital through sustaining “social order through social as well as biological reproduction.” Therefore, the family was both a universal norm and virtuous privilege that allowed the accumulation, transmission, and utilization of capital between and across generations. Reay (2000, p. 570) explained the importance of family in cultural capital by highlighting how the emotional capital contained in familial social capital provided “the link between individual and class trajectory” (Reay, 2004, p. 59). Emotional labor is an important aspect of family involving an individual (usually women) “carrying” reproductive decision-making and family life in their specific socio-economic context (Hall, 2022). Cottingham (2016, p. 452) defines emotional capital as a “tripartite concept composed of emotion-based knowledge, management skills, and capacities to feel,” and outlines that emotional capital is not exclusively feminine or gender neutral. The possession of capital gives one legitimacy, recognition, and status, and can be converted, exchanged, transacted, and transferred from and between different arenas (Crew, 2020). Capitals are therefore, “not fixed nor determined but relational” (Burke, 2016, p. 8).

Critics assert that Bourdieu's view of embodiment reinforces or denies social class distinctions (Neveu, 2018). Conversely, other scholars (Skeggs, 2001; Schwarz, 2010; Thatcher et al., 2016) argue that Bourdieu's typology acknowledges the embodied dimensions of capital and the interplay between individual and social structure, encapsulated by the phrase, “The body is in the social world, but the social world is in the body” (Bourdieu and Wacquant, 1992, p. 73). Various capitals account for the complexity of intimate relationships: “bodily,” “erotic,” “corporeal,” “sexual,” and “physical” (Bourdieu, 1984; Shilling, 2004; Martin and George, 2006; Hakim, 2010; Schwarz, 2010). Bourdieu (1977) emphasized the study of both habitus and “bodily hexis”—the experience of living in one's body (Inhorn, 2007, 39). Martin and George (2006, p. 125) argue that bodily hexis can yield social capital, noting that “sexual capital is wholly a matter of the body and its bearing or hexis” (Martin and George, p. 128). Hakim's (2010) concept of “erotic capital” is an exception and consisted of beauty, sexual attractiveness, social skills, liveliness, social presentation, sexual competence and fertility. Hakim characterizes the latter as “reproductive capital,” as solely applied to women and less valued in “modern” societies than “agricultural” ones (Hakim, 2010, p. 501). Critically, Hakim only referred to RC once and did not explore its intersection with all other forms of capital or habitus. Acknowledging Hakim, Hadley (2015, 2021a) identified RC (including aging and biological capital to the four original capitals) as a way to understand the complex intersection of factors in male childlessness over the lifecourse. Chen (2025) offers RC (formed by economic, cultural, social and symbolic capital) as a way of understanding Taiwanese gay fathers and fathers-to-be investment in TCS. In this piece we further develop RC as a concept by examining the relationships and intersections among capital(s), habitus, and hexis.

The global marketization of “sex cells” (Almeling, 2011) has been identified as a form of neoliberal eugenics (de Melo-Martin and Goering, 2022) through the reinforcing of the commodification and exploitation of low-income women by high-income consumers (Pande, 2015; Rudrappa, 2015; Manali and Avishek, 2020; Smietana et al., 2021; Pande, 2022; Tober, 2024) and the increase in infant and surrogate trafficking (United Nations, 2019; Crawshaw and Van Den Akker, 2021). Gammeltoft and Wahlberg (2014, p. 201) reason newer reprogenetic techniques (e.g., gene selection/manipulation, Coco, 2014) do not treat infertility but, “prevent or allow the birth of certain kinds of children.” We argue that not fulfilling the pronatalist norms of social, cultural, and symbolic capital results in a form of symbolic violence. Not only is this “an affective violence” (Threadgold, 2020, p. 103), but it also includes eugenic, euphemistic, and obstretric violence. Using Bourdieu's (1987) concept of “symbolic power” (including symbolic violence), Link and Phelan (2014, p. 25) argue that “stigma power” is concealed in “taken-for-granted aspects of culture and thereby hidden or “misrecognized” by both the people causing the harm and by those being harmed.” The inherent sociocultural and structural forces of stigmatization on health behavior and access to healthcare are well-established (Stangl et al., 2019).

The correlation between ill health, low income, social inequalities, and structural disadvantages has been widely recognized (Kelly and Green, 2019; Yopo Díaz and Watkins, 2025). Kriznik and Kelly (2016) described the intersection of health and social inequalities with social factors (class, poverty, pollution) and biological mechanisms (epigenetics: controlling gene expression via deoxyribonucleic acid (DNA) modification) as “biological capital.” The relationship between biology and biography is demonstrated through the strong connection between life-course development, health inequalities, wellbeing, epigenetics, maternal environments, and reproduction (Kriznik, 2016; Vineis and Kelly-Irving, 2019; Yopo Díaz and Watkins, 2025). During neonatal, adolescent, and adult stages (Tomova and Carroll, 2019), reproductive and health problems can influence fertility through “exposed biologies” (Wahlberg, 2018) where DNA damage is caused by the toxic effects of harmful environments (Tomova and Carroll, 2019; Vineis and Kelly-Irving, 2019).

The working environment is one in which individuals and organizations negotiate the intersection of informal and formal pronatalist structures—from an individual's identity, self-identity, and way-of-being-in-the-world to everyday accepted and expected socio-cultural norms, such as maternity and paternity laws and policies. Mccracken et al. (2017, p. 60) emphasizes the importance of social capital in the work environment: “the bond which ties all other forms of capital together.” RC intersects with various combinations of economic, social, and cultural capital that facilitate or inhibit ways of being-in-the-world, including the field(s) and subfield(s) of the workplace and wider social arenas with their discrete, diverse, distinct, and dedicated conventions, practices, and sociocultural norms. Having established the relationship between inequalities, social distinctions, and biological and other forms of capital, the next section details the methods used in this study.

## 3 Materials and methods

This article focuses exclusively on the empirical data derived from narrative interviews conducted as part of a 2-year (2020–22) research study investigating individuals' experiences in the workplace while undergoing fertility treatment. The project comprised three primary components: biographical narrative interviews with employees who have firsthand experience with ART (the central focus of this article), semi-structured interviews with managers and fertility counselors, and a desktop review of online resources.

### 3.1 The sample

Participant recruitment was through open calls on social media via professional, personal, and fertility-related organizations and networks and included snowballing by participants. The definition of complex fertility journeys was intentionally broad, leaving it open for participants to decide what it meant to them. The recruitment material referenced “deciding if/when to try for children; trying for children; fertility tests; fertility treatment; pregnancy loss; secondary infertility and involuntary childlessness.” The study was open to any nationality and participants could work (or have worked at some point during their fertility journey) in any sector or occupation. The participants could be at any point in their fertility journey. Exclusion criteria were anyone under the age of 18 years and those who were not proficient in English. Given the 2-year length of the study, the aim was to recruit 60 participants, as this was deemed sufficient for an exploratory study of this issue and also reached the criterion of data saturation. However, the response was greater than anticipated. Due to COVID-19 restrictions, most interviews were conducted using Microsoft Teams or Zoom. Using theoretical sampling, 80 biographical narrative interviews (67 women and 13 men) were conducted. Consequently, the sample was predominantly women (see limitations), which is a common bias in fertility treatment studies (Lloyd, 1996; Inhorn et al., 2009; Hadley and Hanley, 2011; Lohan, 2015; Hadley, 2024). Seventy-three lived and worked in the UK, with the remainder coming from Australia (a married couple), Cyprus (one), Luxemburg (one), the Netherlands (one), and the USA (two; unrelated). The sample included 69 people in heterosexual partnerships and six people in same-sex relationships. Five women pursued solo motherhood (one bisexual, one lesbian, and three heterosexuals). At the time of the interview, 37 participants were accessing ART treatment, and 43 participants had completed the treatment: 21 with children and 22 childless. Some participants did not pursue treatment because of age, cost, IVF effects, and/or perinatal loss. All participants quoted in this piece received ART treatment. Table 1 details their social characteristics, points in their fertility journey, gender, sexual orientation, job (sector, role), and Standard Occupational Classification (SOC; Office for National Statistics, 2023). SOC indicates the participants' socioeconomic grouping at the time of the interview and identifies the level of education and skills required for a job. Some participants in the UK initially received NHS treatment, although most were either paying for or paid for treatment. Phil (Australia) and Donna (USA) had treatment through health-insurance. The ethics committee of the Manchester Metropolitan University approved this study (case 20547). Informed consent was obtained prior to the interview; pseudonyms were used to ensure confidentiality and are used in this article.

**Table 1:** Characteristics of quoted participants.

**Name**	**Gender**	**Sexual orientation**	**Relationship status**	**Age treatment started**	**Children at the time of interview**	**Fertility journey ended**	**Job: sector, role**	**Standard occupational classification (SOR)**
Alan	Male	Heterosexual	Married	40+	Yes	Yes	Public sector, academic	2. Professional
Brittany	Female	Heterosexual	Married	30–34	No	No	Public sector, administrator	4. Administrative and secretarial
Chris	Male	Heterosexual	Married	DNR	Yes	Yes	Private sector, consultant engineer	2. Professional
Donna	Female	Heterosexual	Married	35–39	Yes	No	Public sector, academic	2. Professional
Felicity	Female	Heterosexual	Married	30–34	No	Yes	Public sector, academic/medical	2. Professional
Gail	Female	Heterosexual	Married	30–34	No	No	Private sector, accounting	4. Administrative and secretarial
Hannah	Female	Lesbian	Married	30–34	Yes	Unsure	Public sector, teacher	2. Professional
Karen	Female	Heterosexual	Married	<30	No	Yes	Third sector, legal assistant	3. Associate technical
Iris	Female	Heterosexual	Partnered	40+	No	Yes	Public sector, social care officer	3. Associate technical
Leona	Female	Heterosexual	Married	<30	Yes	No	Public sector, school safeguarding officer	3. Associate technical
Linda	Female	Heterosexual	Partnered	35–39	No	Yes	Public sector, teacher	2. Professional
Michaela	Female	Heterosexual	Married	35–39	No	Unsure	Public sector, physiotherapist	2. Professional
Phil	Male	Heterosexual	Married	35–39	No	Yes	Public sector, manager	1. Manager
Queenie	Female	Heterosexual	Married	DNR	No	No	Public sector, teacher	2. Professional
Rita	Female	Heterosexual	Married	35–39	No	No	Public sector, HR officer	3. Associate technical
Sophie	Female	Heterosexual	Married	35–39	No	No	Public sector, medical secretary	4. Administrative and secretarial
Tony	Male	Heterosexual	Married	40+	Yes	Yes	Public sector, manager	1. Manager
Tracey	Female	Heterosexual	Single	35–39	Yes	Yes	Private sector, HR manager	1. Manager
Violet	Female	Heterosexual	Married	30–34	No	Yes	Public sector, physiotherapist	2. Professional

### 3.2 Data gathering

The biographical narrative interview approach in Wengraf's Biographical Narrative Interpretive Method (BNIM; Wengraf, 2011) was used to comprehend both the individual and social contexts of the participants' experiences. Narrative interviews are an established method for understanding individuals' experiences on sensitive topics in latent, hard-to-reach, vulnerable, and/or marginalized groups (Liamputtong, 2007; Hadley, 2021a). Two important features of the BNIM approach are the emphasis on eliciting narratives by contextualizing experiences in relation to the past, present, and future. Second, focusing on the biographical narrative by keeping it within the participant's frame of reference. The interviewers (Mumford and Wilkinson) began each interview with an open question designed to generate narratives (Wengraf, 2011). Participants were asked to describe their experiences of their complex fertility journey and how this intersected with their employment, starting wherever they chose and taking as long as they needed. This allowed each participant to identify and explain important events and experiences. Following the opening narrative, participants were asked to elaborate on the elements of their story to provide more detail and/or ask additional questions, as appropriate. These covered things like the impact of fertility treatment on them at work, disclosure in the workplace, support desired and received, and financial issues. Focusing on the participants' stories, the researchers' subsequent questions formed participant-led narratives which revealed extremely emotional and moving accounts. Interviews were audio-recorded, typically lasting 60–90 min, and professionally transcribed.

### 3.3 Data analysis

Hadley undertook a qualitative analysis (hosted on Nvivo12), engaging in a latent thematic analysis (Braun et al., 2013) which focused on understanding each participant's experience in relation to a broader social context. Thematic analysis works both to reflect reality and to explore the surface of “reality” (Braun and Clarke, 2006, p. 81). Additionally, thematic analysis was inductive because the identified themes were closely connected to the data. The initial codes were generated through iterative coding rounds. Provisional themes were created by examining the initial codes, links, and themes. Provisional themes were used to shape candidate themes and structure the main themes (Braun et al., 2013). The analysis revealed five main themes: (a) economic capital; (b) cultural capital; (c) social capital; (d) symbolic capital; and (e) reproductive capital.

## 4 Results

Participants' experiences of undergoing fertility treatment in the workplace context show how reproductive capital intersects with other forms of capital (economic, social, cultural, and symbolic). The data illustrates Bourdieu's concepts of habitus and field, showing how reproductive status shapes one's position and experiences in the workplace field. Men's and women's narratives highlight how economic capital enables access to fertility treatment, whereas a lack of economic resources can constrain reproductive choices. Overall, our findings support Bourdieu's framework by illuminating the complex interplay between different forms of capital, and how they reflect inequalities related to reproduction in the workplace. In the following sections, we examine how RC intersects with biological, social (focusing on “symbolic violence”) and economic capital.

### 4.1 Reproductive capital

Reproduction is highly prized in most societies and is culturally reinforced. For example, in the field(s) of the workplace, “natural” pregnancy triggers the interaction of RC between habitus and biological and the four original capitals. Tony observes the dissonance between how men think and feel about fertility and fatherhood and the available social narratives:

“I think men can…be accused of being a bit matter of fact about fertility… we come across differently to how we feel about it - it's very, it's very important to us.”

Tony demonstrated how masculine stereotypes of invincibility and unemotionality restrict men's expressiveness of vulnerability, particularly in social environments (Daniels, 2006; Hadley and Hanley, 2011; Hadley, 2021a,b). Queenie illustrated how the RC of a pregnant colleague actuated rights and status which impacted Queenie's workload, but also reflected the non-recognition of her and others' ART fertility journey:

“You have to bend over backwards for women who are actually pregnant … where's all that for women who are going through fertility treatment?”

Queenie highlighted the primacy of RC at agentic and structural levels. Likewise, in undergoing ART, Rita explained how not being able to draw on biological and RC impacts the other capitals, fields, and habitus. Both Rita and Tony emphasized the feeling of being part of a process during their respective IVF and Intracytoplasmic Sperm Injection (ICSI) journeys:

“One of the hardest things about this fertility journey is that you have very little control over what's happening to you…your body…the process…the decisions that are made.” (Rita)

“How you feel…it's a massive impact, and you feel like you're being carried along really…it's like a process you're in. I felt that.” (Tony)

Rita's and Tony's experiences demonstrated the challenge to identity and self-concept that ART treatment has: an existential question that permeates all settings and relationships. Infertility is linked to biological capital and intersects other forms of capital. The diminutive impact of not fulfilling personal and social status reveals how capital relates to the covert and overt dynamics of their interconnection. The following sections examine the relationship between RC and other forms of capital.

### 4.2 Biological capital

Bourdieu (1986) viewed family as central to cultural capital and explained that a person's cultural capital is limited to “appropriating capacities of an individual agent; it declines and dies with its bearer (with his biological capacity, his memory, etc.).” Although an individual's cultural capital ends at death (apart from legacy items), biological capital may continue through their offspring. In addition, cryopreservation provides an opportunity for a deceased person's stored gametes to create a child (Deech and Smajdor, 2007). Thus, biological capital interacts with RC and cultural, economic, social, and symbolic capitals. Biological capital can intersect with RC in several ways, with many people's reproductive resources being affected by hereditary genetics or other health issues. Kriznik (2016) demonstrated social factors such as class, environment, and diet can influence baby *in-utero*. The participants referenced the following underlying health issues:

“We were having IVF for PGD [Pre-implantation genetic diagnosis] because my husband is registered blind…we didn't want to pass it on.” (Brittany)

“I was diagnosed in my teens with polycystic ovaries… I was told… I would probably find it difficult to conceive… the advice… was that when I started to want to try for a baby to seek help sooner rather than later.” (Donna)

Genetic predispositions and health issues not only affect biological reproduction and RC but also impact the future accumulation of cultural, social, and symbolic capital for individuals and families. Similarly, Gail's experience highlighted how the intersection between aging, biological, economic, emotional, social, and symbolic capital, and habitus impacted her mental health.

“I hit my 40th birthday, it was big…because paying for it ourselves…we knew the chances…with both of our problems were going to be low anyway. I…went into major depression because of not going to be a mum.”

Gail's reactions show how her reproductive capital intersects not only with economic, social, and symbolic capital, but also highlights her perceptions and dispositions toward reproduction. Bourdieu (1986) argued social capital was “a continuous series of exchanges in which recognition is endlessly affirmed and reaffirmed.” In Gail's example, we can see how not being affirmed impacted her self-view and her partner. The participants' personal stories support research findings identifying a wide range of factors that influence childlessness (in addition to biological and health issues), including choice of partner, relationship dynamics, and the timing of relationship formation and ending. Tony's experience reveals the dynamics between biological and other capitals. In a previous relationship, Tony had undergone vasectomy after becoming a father. In his current relationship, his partner wanted children. Consequently, Tony had an unsuccessful vasectomy reversal before ICSI resulted in successful IVF treatment:

“We had the family…the house…the careers…I had the…vasectomy…that was successful…I was infertile. That relationship broke down…I met [new partner]. Fertility was an issue … I knew it was my fault. I had vasectomy reversal. It was quite painful…to find that it hadn't worked that was hard…We decided to try…surgical sperm removal [ICSI]…they took it directly from me, in surgery…”

Tony's fertility journey reveals the intersection between RC, biological, cultural, economic, social and symbolic capitals. His story shows the dynamic interaction between RC, fluidity in family forms (Morgan, 2011) and significance of relationships across time. The following section explores the relationship between social capital and other types of capital.

### 4.3 Social capital and workplace dynamics

The workplace is a key site for the accumulation, exchange, transmission, and utilization of economic, cultural, social, and symbolic capital through social groups, networks, obligations, and individual relationships. Alongside the disadvantages faced by pregnant women and marginalized parents (Fox and Quinn, 2014; Tarrant, 2021; Owens, 2022), in interpersonal workplace interactions, childfree and childless workers can be viewed as “deviant to pronatalist ideologies that workers carry” (Mullins, 2018b, p. 165). Working fathers and mothers have a higher status and are more trusted by coworkers than those without children (Zhang and Soderberg, 2023). Everyday interactions reflect the nuances of the pronatalist agenda and the emotional depth of not accessing one's RC. Workplace announcements of pregnancy or birth had a deleterious effect on many participants. Gail, Karen and Felicity highlight the emotional labor involved in negotiating the social disenfranchisement of not fitting the pronatalist habitus (Exley and Letherby, 2001). In addition, Karen reflected on the pervasiveness of the “ideal” parental narrative and highlighted the ubiquity of childrearing as mainly associated with women:

“Pregnancy announcements are a big thing … it feels like everyone around you is pregnant… you cannot escape it.” (Gail)

“People talk about their baby photos and stuff… I couldn't get my head around that it was the men talking about their babies and it was like, ‘Oh, it's not just the women, it's the men.”' (Karen)

“It's…a lonely place when a lot of people are having babies…and you're not … your network gets a bit smaller…Until it's nobody.” (Felicity)

Felicity observed that the response to COVID-19 had made working-from-home and video meetings acceptable working practice. However, virtual meetings became an opportunity for parents to include their children not only in the virtual workspace, but also in their homes.

“People are bringing new-born babies to Zoom calls… I've had, ‘Oh, let me show you…”'

For the participants, the impact of colleagues' familial demands on working practices highlighted a change in cultural and symbolic capital; the attainment of parental status resulted in a difference in working practices. This results in colleagues becoming familial support by proxy.

“…people just assume with kids that they can leave early or do this or do that.” (Karen)

Other locations were sites where not converting to RC led to the diminution of other capital. Felicity explains how not being a mother affects her social network.

“It is difficult to make friends with women…most of them have children…I see my sisters with all their friends…I envy how easy it is for them to make friends…we just don't have that available to us.”

Felicity's experience demonstrates the dynamics between aging, emotional, RC, and social and symbolic capital in interpersonal social fields (Ramsay and Letherby, 2006). Changes in demographic trends have led to an increase in the number of grandparents and a decrease in the number of grandchildren (Timonen and Arber, 2012). Chris reflected on how disruption from the ideal life trajectory continues across the lifecourse.

“And that isn't really going to go away either…our friends are one day going to be grandparents as well. So, it's never really going to stop.”

Social relationships and social identity are intrinsically linked and are extremely important to health and wellbeing. Consequently, activating RC involves the accumulation, exchange, and utilization of social, cultural, and symbolic capital. Participants' experiences revealed the complex intersections between capital, habitus, and hexis when reproductive dispositions were disrupted. Those who experience unwanted parenthood also face similar difficulties. While this section focused on personal interactions, the next section focuses on the structural implications of RC.

### 4.4 Symbolic capital and symbolic violence

The participants' narratives established the structural (legislation and policy) and interpersonal authentication (presents and parties) that fulfilled the pronatalist procreative imperative. Moreover, they clearly demonstrated the impact of *not* fulfilling this status. Thus, the utilization of biological (and, in some cases, economic and social) capital results in the accumulation of cultural and symbolic capital.

### 4.5 The workings of symbolic violence in inequalities

Bourdieu argued that suffering caused by “social hierarchies and social inequality” (Schubert, 2008, p. 183) produced and maintained through social domination. One such form of domination was symbolic violence which “is only exerted through the communication in which it is disguised” (Bourdieu, 1977, p. 237). Bourdieu's concept of symbolic violence explains how social hierarchies are maintained, revealing how power acts through arbitrary culture to maintain dominance and exclude the dominated by inculcating ways of thinking, expressions, and ways of meaning in fields and habitus (Bourdieu, 2008; Grenfell, 2008). Not attaining the desired distinction (socially approved status) induces the soft power of symbolic violence. Symbolic violence is exercised in the microcosm of the everyday and is often misrecognized as natural and legitimate (Thapar-Björkert et al., 2016) compared with other types of violence, that is, economic and physical. However, it can be brutal and has serious effects on mental and physical health (Schubert, 2008). Consequently, Bourdieu (2002, p. 171) argues people are unconsciously compliant because “it is itself the effect of a power…beliefs which make one sensitive to certain public manifestations, such as public representations of power.” As noted previously, violences associated with reproduction (including “eugenic,” “euphemistic,” and “obstetric”) are more experienced by marginalized groups disadvantaged by age, class, ethnicity, gender, and race. The everyday workplace environment reinforces social and cultural hierarchies through the normalization of symbolic violence through the perpetuation of gender inequalities. Hannah highlighted inequalities in national policy and clinical practice through the discrimination of same-sex couples:

“…with a heterosexual couple, you've just got to declare to your doctor, ‘I've been trying for a long time.' But with a same-sex couple…you have got to … have six attempts of insemination before they can see that.”

The power embedded in legislation and employer policies is also communicated through other forms of inequality. Although many participants had supportive line managers, others did not:

“I once had to argue with my manager that the three or four days off I'd had after a miscarriage was pregnancy related.” (Iris)

Iris's emotional distress uncovers one of the many challenges in everyday workplace interactions: the lack of understanding and accommodation of the losses experienced by people undergoing fertility treatment. Here, RC affected Iris's emotional, social, and symbolic capital. The emotional responses reported were frequently grounded in the microcosm of social interactions, where the ubiquity of parenthood generated feelings of exclusion.

“It sometimes feels like if you have a child…kind of green light to… ‘I need to do this' ‘I need to leave early.' But if you don't have kids, it feels like you can't ask those things.” (Linda)

Linda's experience highlight the impact of not attaining the desired social status, leading to a lack of cultural and social capital. The nuances involved in symbolic violence against those who do not conform to an ideal social status range from the macro (Hannah and Iris) to the micro (Linda). Although symbolic violence is latent within social interactions, Southerton (2011) asserts that once it is recognized, it ceases to become symbolic violence.

“I'm not a victim; I don't want to be treated differently or specially … all I want is just to be acknowledged that this is what you've been through…” (Leona)

The participants' experiences highlight how the primacy of the biological imperative is structurally embedded in the socio-cultural pronatalist doctrine, discourse, and practice. Not achieving or declining to participate in an ideal symbolic status invokes consequences at the agentic and structural levels. The study reveals the complex ways RC and other forms of capital intersect, shaping workplace dynamics and individual behavior. This highlights the need for more inclusive policies and cultural shifts to recognize diverse reproductive journeys.

### 4.6 Economic capital

The substantial costs associated with ART procedures, combined with widespread misunderstandings regarding their efficacy, result in considerable financial and psychological challenges for numerous individuals and couples. Access to ART depends on many factors, including location, health delivery regime, policy, and personal situation. In the UK, ART is available on the NHS, although admission is not straightforward and varies from one area to another. Consequently, NHS ART treatment is often labeled as a “postcode lottery” (Meredith, 2022) and private ART treatment is extremely expensive. In countries with health insurance, ART options are a limited part of an employer package, a costly add-on, or not included. Following unsuccessful ART, Phil viewed the fertility sector as focused on capital accumulation (bio-capitalism):

“You do get the sense that the IVF industry's unit of production is number of treatments rather than necessarily number of best decisions made about fertility.”

The focus on quantity over quality of treatment exacerbates financial and other stressors on people's capital and habitus, who may pursue multiple costly rounds based on inflated expectations of success. It is well-established that people misrecognize the effectiveness of ART and many believe that IVF easily mitigates any delay in parenthood (Thompson and Lee, 2011; Daniluk and Koert, 2013). In addition to IVF, cryopreservation (egg freezing) has been promoted as a solution to prevent career disruption and/or find a suitable partner (Baldwin, 2018; Inhorn, 2023; Tober, 2024). The misconception that ART can easily overcome age-related fertility decline leads some to delay childbearing and become unaware of its potential financial, health, identity, and social implications. Sophie reflects on her colleague's view of ART:

“Some women… assume that IVF is the answer to everything… ‘I'm going to freeze my eggs, because then there'll be a 30-year-old's egg.' But you need the funding to do that.”

Sophie's observations highlight two factors. The first is the success of the fertility industry's campaign for mainstream egg freezing (Van De Wiel, 2020; Tober, 2024). Van De Wiel (2020, p. 307) argues that “egg freezing is both an infertility treatment for the fertile and a fertility treatment for the infertile.” The optimistic promotion of ART often clashes with the economic realities of those pursuing it. Second, the widespread belief in the efficacy of ART. The situations described above highlight the dynamics in the accumulation and diminution of and between aging, biological, cultural, economic, reproductive, and symbolic capital. Given the unpublicized high rate of unsuccessful treatment (“euphemised violence”), the invasive nature of treatment (“obstetric violence”), and inequalities of access to treatment, there is a compelling case for acknowledging the symbolic violence inherent in ART. The intersection of economic capital and reproductive choices is further complicated by socioeconomic disparities in access to and utilization of ART. Reay (2004, p. 58) argues that Bourdieu focuses on the middle and upper classes to demonstrate the various forms, distribution, and utilization of capital. In Italy, economic uncertainty (e.g., insecure first jobs) is a factor in fertility postponement by highly educated women and the lowest-educated men (Vignoli et al., 2020). Jensen (2016) identified how upper-middle-class men deliberated with their partners over the timing of the first birth for over a year. However, working-class men had few secure relationships, resulting in fewer discussions concerning parenthood. Moreover, young working-class solo parents are often stigmatized as “feckless” fathers and “irresponsible” mothers (Tarrant, 2021; Owens, 2022). The following accounts demonstrate how participants used their economic, cultural, and social capital to accumulate RC and symbolic capital:

“‘We borrowed the money so we could do it quickly …' because you do feel like you're buying something. You are buying something.” (Tony)

“We probably spent £60,000 to £70,000 …my parents…helped out with a couple of rounds. My brother…helped out with one round…most of it was self-funded… that did take that stress out of it.” (Violet)

“It was important that I kept my job…we'd taken out that mortgage to pay for that treatment…So this is my investment [shows child]…all those little financial points were…planned…we were very fortunate…we could make that choice.” (Alan)

Significant financial investments reflect both the desperation felt by many facing fertility issues, and the relationship between economic, cultural, social, and symbolic capital and habitus. Participants whose treatment had been successful reflected on the consequences if that had not been the outcome.

“It sounds terrible, but you think, if this doesn't work, we're going to be paying for this for the next 25 years…There's that whole sense of, it's got to succeed otherwise what was it all for? It's quite a gig.” (Alan)

“I've got a good corporate job…I can afford to pay for IVF. A lot of the people I know…it's really difficult…to pay for that treatment.” (Tracey)

These participants' reflections on ART economics highlight how economic inequalities impact access to fertility treatment and reproductive choices. Illustrating how structurally embedded the disadvantages faced by minority groups are, Hannah relates how she and her partner struggled against gender bias in a heteronormative system:

“a same-sex couple…you have got to…have six attempts of insemination… insemination in the UK is about £1500 a go… quite a bit of money.”

Hannah's experience illustrates the intersection between biological, economic, social, and symbolic capital through the discursive restraints of heteronormativity, highlighting issues faced by marginalized and minority communities (Pande, 2015, 2022). In addition to the economic capital needed to afford treatment, there are costs involved in how people manage their paid employment environment before, during, and after the treatment. The costs involved are not only related to economic capital but also to cultural, social, and symbolic capital, including obstetric violence. Michaela outlines the pressure to balance economic capital and RC:

“My bosses have said, “Did I want to reduce my hours for a period of time?” But we've got an IVF bill to pay.”

The participants' experiences demonstrate how RC directly intersects cultural, economic, social, and symbolic capital. For example, through the utilization of familial networks (social and cultural capital), for others, through their own economic capital.

The complex relationship between economic and reproductive capital reveals how misconceptions about ART's effectiveness can lead to not only substantial financial strain but also pressure social capital and challenge an individual's habitus and hexis. By incorporating the concept of reproductive capital into workplace policies, organizations can create more inclusive environments that recognize and value the diverse experiences and responsibilities of their employees. This approach not only promotes gender equality, but also enhances employee satisfaction, retention, and overall productivity. This section identified the intersection of RC with other capitals, and in relation to habitus and fields. The following section contains the discussion, limitations and conclusion.

## 5 Discussion

By using a theoretical model to conceptualize reproductive preferences as habitus, we believe that based on the evidence presented in this paper, there are grounds for pronatalism to be seen as habitus because of its effect on all sexes and genders: “The habitus is not only a structuring structure which organizes practices and the perception of practices but also a structured structure” (Bourdieu, 1984, p. 166). The participants' experience of fitting (or not) personal and societal expectations/norms revealed the relationship between various forms of capital, habitus, and field. The Bourdieusian perspective demonstrates the symbiotic relationship between capital and habitus; each simultaneously interacts with and shapes the other. Integral to habitus is a bodily hexis and its intersection with social capital (Martin and George, 2006). In this study, we show the complex interaction between capital, hexis, and habitus involving the accumulation, diminution, transmission, and utilization of capital. This is demonstrated by the intersections between biological capital and RC. The former is affected by factors such as genetic history, location, trauma, and other forms of capital, such as class and education. These may impact RC, making conception less likely. Similarly, hormonal and cellular changes over time (i.e., puberty, menopause, andropause) result in changes to hexis, habitus, reproduction and other forms of capital. The participants narratives demonstrated how RC intersects with other forms of capital and concepts

RC is intrinsically linked to biological capital, which is an individual's biological capacity for reproduction. Tony's fertility journey/career (involving biological fatherhood, vasectomy, vasectomy reversal, ICSI) and his declaration of his baby as “my investment” sums up the direct connection between RC and the rhizomatic relationship with biological, cultural, economic, social, and symbolic capital across the life course. Biological factors (e.g., Brittany's partner's genetic condition and Donna's polycystic ovary syndrome) directly impacted their RC–not only their reproductive career but also their accumulation of cultural, social, and symbolic capital. For the most part, biological capital “declines and dies with its bearer” (Bourdieu, 1986). However, cryopreservation technologies mean that for some, their RC can continue. Gail's inability to become a mother deeply affected her mental health, identity, and way-of-being-in-the-world, all interconnected with economic, cultural, social, and symbolic capital. Many participants, such as Alan, Tony, and Violet, took out loans or drew on familial financial support to fund the treatment, highlighting the connection between economic capital. Phil's perspective on the fertility industry as “bio-capitalism” focused on “number of treatments rather than necessarily number of best decisions” highlights economic pressure and potential for exploitation. Moreover, the misconception that ART can easily overcome age-related fertility decline, as noted by Sophie, often leads to delayed childbearing without full awareness of its significant financial, health, identity, and social implications, further illustrating the intersection of economic capital with biological, social, and cultural capital.

The study showed how RC directly interacts with social and cultural capital, influencing an individual's identity relationships and status in society in all environments, including the workplace. Pervasive pronatalist ideologies in the workplace mean that working parents often gain higher status and trust from colleagues. The emotional impact of colleagues' pregnancy announcements on participants undergoing ART highlights the non-recognition of their fertility journeys and the struggle to “activate” their RC. Queenie's observation about having to “bend over backwards” for pregnant colleagues' while her own ART journey received no such consideration illustrates the primacy of RC at both individual and structural levels. Felicity's awareness of changes in social network size and shape related to peers becoming parents demonstrated how not a non-active RC can lead to a diminution of social capital. Similarly, Chris's reflection on friends becoming grandparents highlighted not only the change in demographics, but the potential impact of childlessness in mid- and later life. This aspect of RC outlines how the influence of reproductive careers, identity, justice, stratification, etc., is active across the life course.

Bourdieu's concept of symbolic violence highlighted the subtle, yet profound harm experienced by individuals who do not conform to pronatalist ideals or who face reproductive challenges. Not fulfilling the “procreative imperative” results in a form of “affective violence” that is often “misrecognized” as natural or legitimate. This is evident in workplace interactions where individuals without children (for whatever reason) or those undergoing ART face stigmatization, discrimination, and exclusion. Examples include Iris having to argue that her miscarriage was “pregnancy-related,” and Linda felt unable to ask for workplace flexibility because she did not have children. Hannah's experience as a lesbian woman struggling to access NHS fertility services due to discriminatory policy identifies how symbolic violence is embedded in legislation and clinical practice, disproportionately affecting marginalized groups. This demonstrates how RC helps to elucidate the structural inequalities faced by minority groups, reinforcing disadvantages based on gender, class, and sexual orientation.

RC has a positive relationship with existing reproductive frameworks in several ways. Social reproduction allows a deeper understanding of the relationship between how reproductive journeys are shaped by inequalities and social structures. RC reveals how inequalities in accessing ART and other resources are stratified by class and economic, social, and other forms of capital, leading to inequalities in reproductive outcomes. RC contributes to the challenges identified by the reproductive justice approach by identifying the economic, social, and cultural barriers faced by marginalized groups and others. RC helps illustrate how reproductive practices and events (for example, ART, childlessness, parenthood) are interconnected biosocial components across the life course situated within specific social and economic contexts. Consequently, RC supports and builds on the concepts of reproductive identity and careers. While Hakim's (2010) concept of “erotic capital” included “reproductive capital” as one of several sub-capitals, this was only applied to women and reported as less valued in “modern societies” than agricultural ones. Furthermore, Hakim did not explore its relationship with the other elements of Bourdieu's framework (Pande, 2015; Smietana et al., 2021; Tober, 2024). Similarly, Chen (2025) utilized a form of RC comprising cultural, economic, social, and symbolic capital in his study of Taiwanese gay fathers and fathers-to-be in transnational commercial surrogacy, highlighting the stratified reproduction issues faced by marginalized and minority communities. By comparison, our form of RC explicitly explores RC's intersection of RC with all other forms of capital, habitus, and hexis. We include aging and biological capital for a more comprehensive understanding of complex factors, such as male childlessness. In addition, we highlight the globalization of the fertility industry, bio-capitalism, financialization of fertility (Van De Wiel, 2020) and ethical implications and inequalities surrounding TCS (Pande, 2015; Smietana et al., 2021; Tober, 2024). We believe that our form of RC allows for a deeper understanding of how reproductive resources and choices are shaped by environmental, individual, social, and structural conditions that either enable or constrain an individual's reproductive journey.

For adults, RC (capacity to become biological parents) provides access to cultural, social, and symbolic capital. In addition, it connects the past, present, and future. The “past” through positive or negative hereditary items that may include genetic issues (biological capital), money/property (economic capital), legacy items (cultural capital) and family situation (cultural, social and symbolic capital). The “present” via negotiating the socio-cultural normatives such as the biosocial clock inherent in pronatalist societies. The “future” by allowing for future genetic continuation and social roles such as grandparenthood (Timonen and Arber, 2012; Hadley, 2018). As such, utilization of RC directly engages with Bourdieu's (1986) description of social capital “a continuous series of exchanges in which recognition is endlessly affirmed and reaffirmed.” It also connects with economic capital, the costs of raising a family against the accumulation of cultural, social, and symbolic capital across the life course. Accumulation, conversion, transmission, and utilization of RC provide social distinction. Not doing so or doing so but not filling socio-cultural ideals may invoke symbolic violence.

We argue that there are opportunities for governments, employers, and human resource professionals to recognize RC and its impact on workers, the working environment, and relationships. One way forward is the adoption and promotion of equitable and inclusive legislation and policies that acknowledge the complex intersection between reproduction, work, and various forms of capital. For example, acknowledging potential triggering or exclusionary situations and expanding provisions/policies to recognize the diverse and fluid nature of “family” structures across the life course.

### 5.1 Limitations

The limitations of this study include the sample which was limited in terms of diversity in class, race, gender, and relationship status. There were more women (67) than men (13), and fewer people not in heterosexual couple relationships. This was despite many efforts to recruit as representative a sample as possible, especially for men. However, there are a number of factors that influence men's participation in sensitive research, including the historical focus on women, data collection bias, masculinity norms, and challenges in recruiting men on sensitive topics (Lloyd, 1996; Inhorn et al., 2009; Hadley and Hanley, 2011; Lohan, 2015; Hadley, 2024). In terms of class, race, gender, and relationship status, this may be linked to several factors, including the impact of COVID-19 on people accessing ART, the sample reflecting the research study's networks, the struggles identified in accessing ART in the UK, and the cost of private treatment for those who experience intersectional disadvantages. Although some participants were from overseas, the majority were White British, professionals in middle-class occupations, and residing in the UK. Consequently, the empirical data speak to a certain aspect of RC: white, professional, employed men and women negotiating their fertility journey. This study is the first to collect data on fertility journeys in relation to the work environment. The concept of RC was drawn from the data. Future research should consider gathering cultural, economic, historical, and social background data to illustrate the complex rhizomatic relationship between RC and other forms of capital. For example, Johnson et al. (2023) collected the number (density) and distinct types (complexity) of reproductive events throughout their lives. Future research could expand the use of RC to understand differently positioned childless people and parents, including black, disabled, indigenous, marginalized, migrant, and low-income people.

## 6 Conclusion

This study addresses the paucity of scholarship surrounding the distinctiveness of reproduction across the life course, specifically in relation to individuals' resources (capital) and ways of being-in-the-world. By applying the lens of RC, this study provides critical insights into the intersection of reproduction with class, disability, gender, and race. RC offers a new framework for understanding the complex rhizomatic relationships among reproduction, individual and cultural environments, and societal structures. This adds a new perspective to the intricate intersections of economic and sociocultural environments, inequality, knowledge, power, reproduction, and technology in people's reproductive journeys. Incorporating aging and biological capital into Bourdieu's original framework provides a deeper view of observed inequality, by illuminating how latent and overt structural and structuring inequalities are contingent on people's reproductive agency. Moreover, RC can be employed as an analytical tool to support other reproductive approaches illuminating as it does, the complex nuances in the relationship between individual agency and structure. This perspective builds on and supports issues of inequality, power, and social norms other reproductive approaches have found.

An important finding was the lack of employment legislation, organization policies, and allowances for fertility treatment, and the underlying relationship between pronatalist ideals and RC. Accordingly, we demonstrated the intersection between RC and social capital in organizational settings. The financial strain associated with ART, along with misunderstandings regarding its efficacy, pose considerable financial and psychological challenges. The observation that many participants had to draw on significant economic resources from personal savings, loans, or familial support to access private treatment underscores how ART costs reinforce existing inequalities. This economic barrier, which disproportionately affects marginalized groups, reflects the marketization of global IVF as an investment opportunity. This raises the question of whether this constitutes “economic eugenics,” where access to reproductive choices is dictated by financial capacity. Those not partaking in the ideal pronatalist imperative are open to symbolic violence that impacts their internal and external worlds across their life course. Most narratives concerning reproduction and the workplace are on family (especially maternity) and focus on career barriers and their effect on capital accumulation. By introducing the concept of RC, the latent and multifaceted inequalities, including discrimination, exclusion, and stigmatization, faced by individuals whose reproductive journeys do not align with idealized pronatalist norms are revealed. This includes not only those undergoing ART but also those who are childless by circumstance or choice, or those in diverse family structures such as same-sex couples or solo parents. These individuals are often subjected to symbolic violence, which affects their internal self-perception and external social interactions throughout their life. This aligns with broader feminist scholarship emphasizing the gendered nature of infertility and cultural norms that disproportionately burden women and marginalize men, often overlooking men's fertility issues. Those who do not conform to the pervasive “ideal” pronatalist imperative are frequently subject to discrimination, exclusion, isolation, misrecognition, mistrust, stereotyping and stigmatization. The experiences shared by the participants underscore the ongoing need for workplaces and society to adopt more inclusive policies and cultural shifts that acknowledge and value the diverse reproductive journeys of all individuals.

## Data Availability

The datasets presented in this article are not readily available because Restrictions related to data protection of personal information. Requests to access the datasets should be directed to Krystal Wilkinson, k.wilkinson@mmu.ac.uk.
